# Indications for early revision surgery for material failure in spinal instrumentation: experience at a level 1 center for spinal surgery – a single-center study

**DOI:** 10.1097/MD.0000000000028410

**Published:** 2021-12-23

**Authors:** Mohammed Banat, Johannes Wach, Abdallah Salemdawod, Gregor Bara, Jasmin Scorzin, Hartmut Vatter

**Affiliations:** Department of Neurosurgery, University of Bonn, Venusberg Campus 1, Building 81, 53127 Bonn, Germany.

**Keywords:** early revision surgery, indication for revision surgery, peak timing of revision, spinal instrumentation

## Abstract

Posterior instrumentation is an established treatment for a range of spinal disorders. Material failure is not uncommon, and the indications for a revision are very heterogeneous. This study aimed to evaluate the indications and timing for early revision spinal surgery due to material failure.

In this retrospective, single-center cohort study, patients underwent spinal posterior instrumentation between January 2017 and July 2019. They were followed up at 3, 12, and 18 months postoperatively. The time of onset of material failure which led to revision surgery was analyzed. In addition, the relationship between the indications for revision surgery and independent variables was examined using a multivariate logistic regression model.

A total of one hundred thirty-five patients were enrolled. Radiolucent zones were found in 30 patients (20%) after 3 months, whereas 48 patients (31%) had radiolucent zones after 12 months. Revision surgery was performed in 13 patients (8.5%). The peak time for revision due to instability was within the first four months of the primary surgery. Multivariate analysis revealed that location, pathology, ASA score, and smoking had no significant impact on the indication for revision surgery, and neither did BMI (*P* = .042). Non-fusion (*P* = .007) and radiolucent zones (*P* = .004), in combination with increased pain (*P* = .006), were predictors for revision.

Our data show that the peak time for early revision of material failure after posterior instrumentation was within the first 4 months of primary surgery. The abnormalities (e.g., radiolucent zones) surrounding the screws without fusion, including persistence of pain, were predictors for revision surgery.

## 1 Introduction

Posterior instrumentation with fusion is an established treatment option for a range of spinal disorders, including trauma, degenerative diseases, infections, and neoplasia.^[1–3]^

Postoperative complications frequently can occur after spinal surgery and represent a challenge in everyday life. Such complications (major or minor) have been reported in the literature, with a rate between 10% and 96% for adult spinal deformity.^[4,5]^ A common typical complication after instrumentation addressed by various authors is loosening of the screws with related material failure, with a range from 1% to 15% up to 63%.^[6,7]^

The large differences and discrepancies in the complication rates described in the literature result from different study designs. These variations consist of study inclusion and exclusion criteria, as well as data collection. For example, many trials are retrospective. Although complication rates are described in many ways in the literature, there are few valid data on the timing of postoperative complications.^[1,8–11]^ The current data regarding revision surgery related to material failure have not been adequately investigated. A study group from the University Hospital of Heidelberg in Germany^[12]^ has summarized the peak timing for complications and revision in spinal surgery. However, the study did not cover all relevant aspects. For example, it is unclear when it is best to repair the material failure and what exactly the indications for revision are. Sanden et al^[13]^ described the radiological indicators for determining radiolucent zones around the screws.

Several factors affect bone fusion, such as bone quality, patient age, and sex.^[14]^ However, fusion materials such as PEEK cages versus other cages, for example, titanium cages, have a different perspective regarding fusion quality and their procedure.^[15]^

Nevertheless, recommendations were not made for several variables, such as clinical parameters.

The study aimed to evaluate the indications and timing for early revision spinal surgery due to material failure.

## 2 Methods

This retrospective, single-center cohort study included all patients with complete data who underwent posterior spinal instrumentation with fusion between January 2017 and July 2019. Follow-up computed tomography (CT) scans were conducted after 3 and 12 months and clinical postoperative evaluations at 3, 12, and 18 months. In case of clinical deterioration, patients are encouraged to report immediately.

The time of onset of material failure, which led to revision surgery, was analyzed. The clinical parameters and demographic data were collected. All patients received a CT scan immediately after the operation, which was used for comparison purposes in the follow-up checks. CT-confirmed abnormalities were documented, in particular the radiolucent zones. The primary surgery was undertaken for instability owing to fracture, tumor, infection, or degenerative deformity. All patients were over 18 years of age. All patients received posterior stabilization – the primary operation – using the same standardized workflow (including median posterior approach and navigation system) and the same instruments (Diplomat system, Signus, Alzenau, Bavaria, Germany) by three neurosurgeons with long years’ experience in spine surgery. Our standard surgical procedure consists of the following steps: median dorsal posterior approach, open transpedicular screw implantation –we do not use percutaneous system-, spinal canal decompression via laminectomy, facetectomy, followed by PEEK cage implantation, and bone implantation for fusion. In cases of spinal tumor (for example, bone metastasis), we implanted screws in the same technique with spinal canal and nerve root decompression in combination with posterior bone fusion, in these cases we have not used cages, but master-graft granules (Firm Medtronic) for the posterior fusion. We used PEEK cages for all posterior instrumentation sub thoracic spine Th 4 in posterior lateral interbody fusion technique. For the thoracic spine, we used additionally costotransversectomy to implant the cages.

During cervical instrumentation and cervico-thoracic junction, we used only posterior fixation and bone fusion from dorsal. For the thoracic spine, we used additionally costotransversectomy to implant the cages.

**Figure 1:**
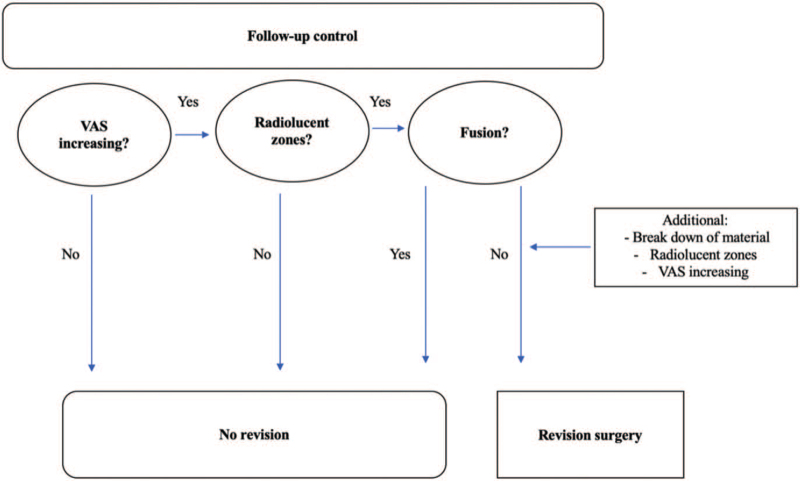
Indications for early revision surgery.

Revision surgery was necessary due to: (1) secondary instability with material breakdown and loss of spinal stability; (2) detection of radiolucent zones around the screws during the CT check; (3) pseudarthrosis in combination with increased back pain. Those factors are our institutional indications for revision surgery. Figure 1 shows our internal algorithm for revision surgery.

During the revision surgery, old material was removed, loosened screws were replaced with thicker screws, and the instrumentation was extended in the appropriate direction. Sonication of the removed screws was undertaken,^[16,17]^ before revision CT and MRI scans were conducted.

### 2.1 Radiological evaluation

Postoperative imaging data were obtained and analyzed by an independent neuroradiologist in accordance with the institutional standards. All patients received CT scans during the postoperative follow-ups. Abnormalities around the screws and signs of instability or fusion/non-fusion were documented.

### 2.2 Statistical analysis

Data were analyzed using IBM SPSS Statistics V22.0 (IBM, Chicago, Illinois, USA). Quantitative, normally distributed data are presented as mean values ± standard deviation (SD), while nonparametric data are summarized by median values [first quartile – third quartile]. Nominal data were analyzed by applying the chi-squared test (two-sided) or, if expected frequencies were <5, Fischer's exact test (two-sided). Data were described as means with SD and frequency (n). A *P* value <.05 was considered significant. Univariate and multivariate analyses were carried out. We also analyzed the correlation between Visual Analog Scale (VAS) and radiolucent zones using a *t*-test, where a *P*-value <.05 was considered statistically significant.

## 3 Results

A total of 153 patients were recruited. The majority of patients (52.3%) underwent a lumbar spine surgery; the most common pathology was a degenerative disease (34%). Table 1 shows the baseline data with univariate analysis.

**Table 1:** Univariate analysis of baseline patient data using Fisher's exact (two-sided) and independent *t*-test.

Total (n = 153)	Revision surgery (n = 13)	No revision surgery (n = 140)	*P*
Age (mean ± SD)	65.46 ± 10.84	67.30 ± 14.20	.65
Gender			.57
Female	7	62	
Male	6	78	
Smoking			.541
Smoker	6	75	
Nonsmoker	7	65	
BMI (kg/m^2^) median [Q1–Q3]	27 [25–29]	26 [25–29]	.079
ASA			.95
ASA 1	0	15	
ASA 2	4	34	
ASA 3	8	71	
ASA 4	1	20	
Localization			.49
Cervical	1	25	
Thoracic	1	24	
Lumbar	10	70	
Another location	1	21	
Pathology			.308
Degenerative	7	45	
Tumor	2	29	
Infection/discitis	3	28	
Trauma	1	38	
Stabilization level			.489
1 level	3	33	
2 levels	3	32	
3 levels and more	7	75	
VAS score, median [Q1–Q3]			
Pre-operative (mean ± SD)	10 [7–10]	9.0 [7–9]	.16
3 months (mean ± SD)	5 [3–9]	4 [1–6]	**.004** ^∗^
12 months (mean ± SD)	4 [2–6]	4 [1–5]	.064
18 months (mean ± SD)	3 [2–6]	4 [0–5]	**.019** ^∗^
Fusion (at 3 months follow-up)			**.002** ^∗^
With fusion	3	47	
Without fusion	10	93	
Radiolucent zones after 3 months			**.005** ^∗^
No	6	117	
Yes	7	23	

ASA = American Society of Anesthesiology, BMI = body mass index, Q1 = first quartile, Q3 = third quartile.SD = standard deviation, VAS = Visual Analog Scale.

∗ Statistically significant.

There was no statistically significant difference in the male-to-female ratio. There was no relevant specificity concerning age and ASA score, nor was there any relevance in relation to smoking or the localization and pathology.

**Figure 2:**
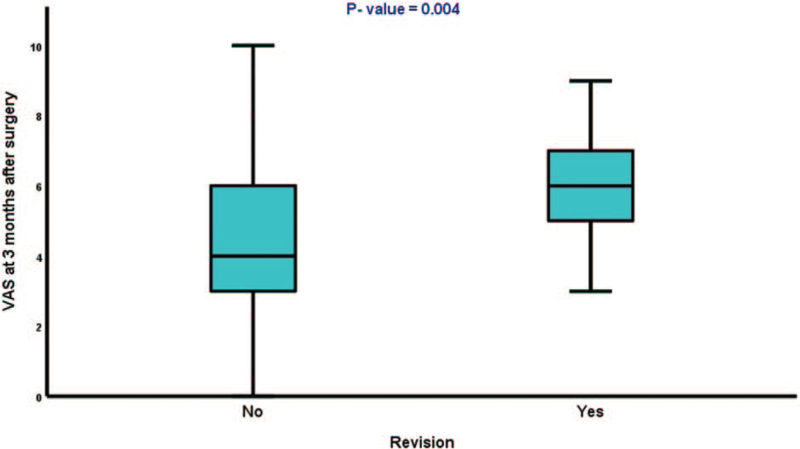
VAS score 3 months after primary surgery; patients who need revision have more pain. VAS = Visual Analog Scale.

BMI is a predictor for revision in the multivariate analysis (*P* = .042). High pain reduction was achieved 3 months after primary surgery among patients who did not need revision surgery, unlike those who did (*P* = .004). Figure 2 shows the different VAS scores in both patient groups. The indication for revision was based on the treatment-refractory pain symptoms and on the image proof of morphological instability (radiolucent zones, break down of material, and non-fusion).

**Figure 3:**
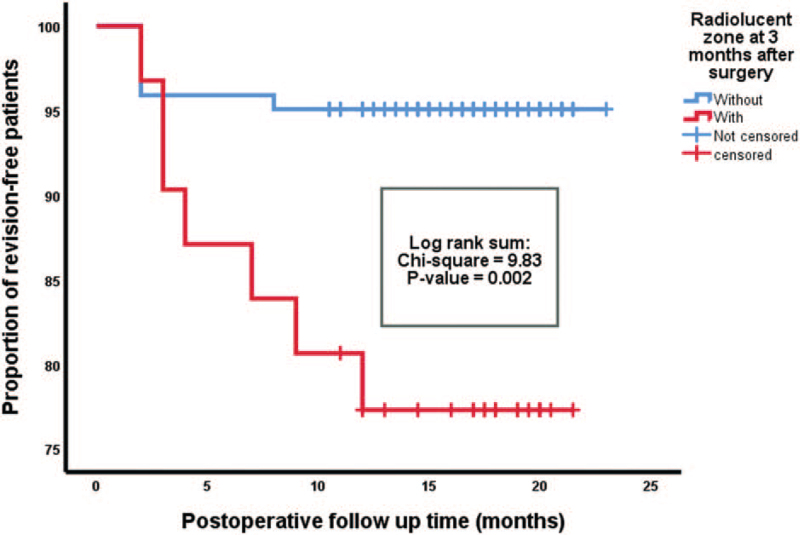
Kaplan–Meier analysis of revision-free probability stratified by radiolucent zones 3 months after spine surgery. Patients with (red) and without (blue) radiolucent zones 3 months after surgery are illustrated. Censored patients (revision-free at last follow-up) are indicated on the curves. The time axis is right-censored at 25 months. *P* = .002 (log-rank test).

Overall, 13 patients (8.5%) had revision surgery about 3 to 4 months after the primary operation (Fig. 3). Radiolucent zones were detected in 30 patients (20%) after 3 months and in 48 patients (31%) after 12 months.

We used a *t*-test to evaluate the relationship between pain reduction and radiolucent zones. Our data show that patients with radiolucent zones had increased pain 3 months after surgery (there were 30 patients with radiolucent zones and 123 patients without): the VAS score (mean ± SD) was 5.2 ± 2.6 in the group with radiolucent zones and 4.1 ± 1.9 in the group without (*P* = .015). The relationship between pain and radiolucent zones was no more significant 12 months after surgery (48 patients with radiolucent zones and 105 patients without): the VAS score (mean ± SD) was 4.3 ± 2.4 in the group with radiolucent zones and 3.6 ± 1.9 in the group without (*P* = .053).

**Figure 4:**
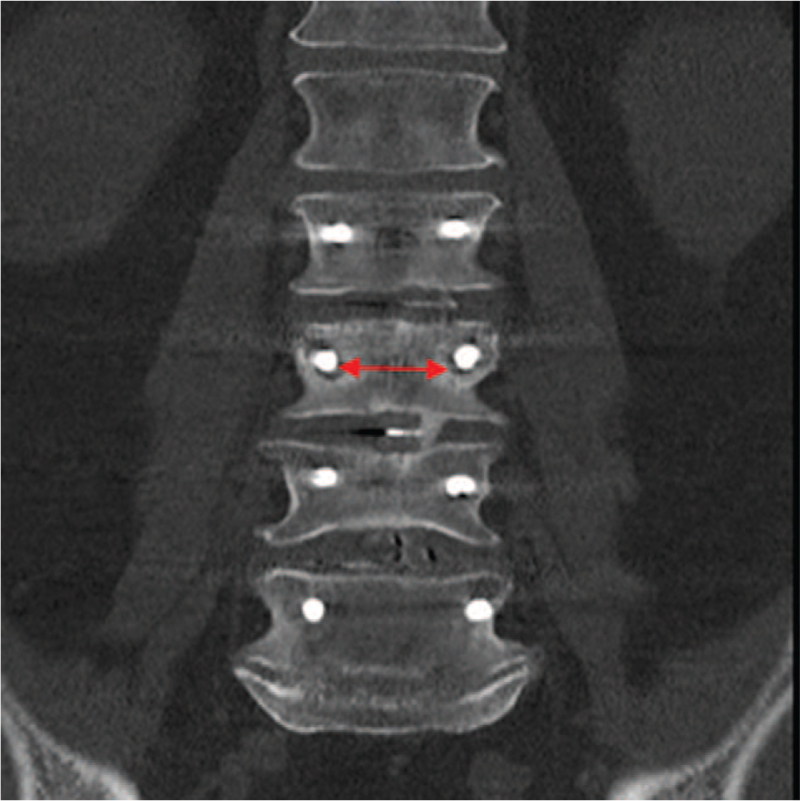
Coronary CT. Radiolucent zone without instability and with fusion of the segments L3-4. Red arrow showing radiolucent zones surrounding the pedicle screws of the lumbar spine L3 at the 12-month follow-up. Procedure in this case: no revision.

**Figure 5:**
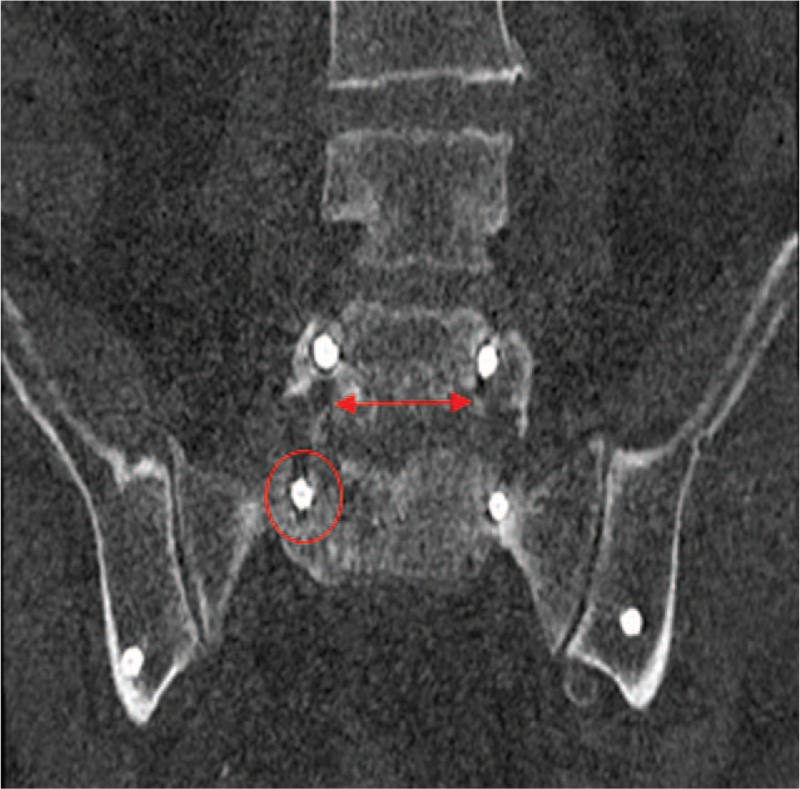
Radiolucent zone with instability and without fusion after L4 – ilium instrumentation at 3-month follow-up. Procedure in this case: revision.

Figure 4 shows a radiolucent zone without instability and with bone fusion (no revision). Figure 5 shows radiolucent zones with instability and without fusion (revision). Multivariate analysis (Table 2) revealed significant predictors for the need for revision, including detection of radiolucent zones (*P* = .004), absence of fusion (*P* = .007), and patients with a high pain VAS score at 3, 12, and 18 months (*P* = .006, *P* = .001, and *P* = .031). Elevated BMI is a negative predictor (*P* = .042).

**Table 2:** Predictors of revision surgery (multivariate logistic regression analysis).

Total (n = 153)	*P*	OR	95% CI	Wald
BMI	.042^∗^	1.7	1.0–2.7	4.1
Radiolucent zone 3	.004^∗^	16.7	2.5–111.1	8.5
Fusion	.007^∗^	15.4	2.1–111.1	7.3
VAS 3 months	.006^∗^	7.6	1.6–14.9	7.6
VAS 12 months	.001^∗^	8.3	2.4–28.6	10.8
VAS 18 months	.031^∗^	4.7	1.1–4.7	4.7

CI = confidence interval, OR = odd ratio, VAS = Visual Analog Scale.

∗ Statistically significant.

Table 3 shows the data for patients who underwent revision surgery. Three patients had partly signs of fusion on the CT scan, but not completely; they had no significant improvement in pain and additionally break down of material; two of them (patient nr. 9 and 10) had radiolucent zones; based on these facts, the revision was carried out.

**Table 3:** Data for patients with revision surgery.

Patient (total 13)	Time of revision (months)	Primary surgery	Radiolucent zone(s)	VAS pre-operative	VAS at revision	Fusion	Level of stabilization	Break down of material	Localization
1	1.5	Discitis	Yes	9	6	No	1	Yes	Lumbar
2	1.8	Degenerative	Yes	9	6	No	1	Yes	Lumbar
3	1.9	Degenerative	Yes	9	6	No	2	Yes	Lumbar
4	2	Trauma	Yes	8	5	No	3	Yes	Cervical
5	2	Discitis	Yes	9	5	No	1	Yes	Lumbar
6	2.3	Degenerative	Yes	8	5	No	5	Yes	Lumbar
7	2.7	Degenerative	No	9	4	No	2	Yes	Lumbar
8	3.3	Tumor	Yes	8	6	Yes	4	Yes	Thoracic
9	3.7	Degenerative	No	9	8	Yes	3	Yes	Lumbar
10	7	Degenerative	No	8	7	Yes	5	Yes	Lumbar
11	7.7	Degenerative	No	7	5	No	3	Yes	Lumbar
12	9	Discitis	No	8	9	No	2	Yes	Lumbar
13	12	Tumor	No	7	7	No	5	Yes	Cervico-thoracic junction

## 4 Discussion

The number of defined complications and the time of onset after posterior instrumentation demonstrate the need to optimize aftercare. Patient data for this study were collected during routine clinical and radiological checks at our spine center at 3, 12, and 18 months postoperatively.

The optimum time for routine follow-up examinations after spine surgery are strongly debated and various recommendations have been made. However, there are no high evidence data that identify the precise point at which material failure peaks after spine surgery.^[4,10,18–23]^

Most patients benefitted from the primary operation and achieved good pain reduction. In our group this was demonstrated after 3 months and persisted for 18 months after the operation. This rate is better than that described in the literature,^[24]^ which we believe is partly due to our multimodal therapy approach after stabilization. This includes appropriate consequently analgesia determined in consultation with the Department of Anesthesiology, as well as early postoperative physiotherapy and later rehabilitation.

One of the most frequently detected complications is delayed material failure, which in our data – unlike Daniels et al^[18]^ – peaks during the first 3 to 4 months after surgery. This finding might be explained by our follow-up schedule, which includes a comparatively early first examination 3 months after surgery. Moreover, we routinely perform CT imaging at the follow-up examinations, which allows a more precise assessment of screw positioning and potential dislocations than X-rays. Pepke et al^[12]^ reported two peaks for postoperative complications. The first peak was observed within the first three months and the second peak in the second year after primary surgery. Another possible reason for the early material failure and pseudarthrosis in our cohort could be that we routinely organize postsurgical rehabilitation starting two weeks after primary surgery. However, the impact of early mobilization after posterior instrumentation on the timing of material failure is not yet known.

The main pathophysiological mechanisms for implants loosening after spinal surgery are often considered to be mechanical causes,^[9,10,19–23,25]^ aseptic or low virulent implant-associated infections, as well as early implant infections.^[17,26,27]^ Indeed, the problem of material failure after posterior stabilization has posed challenges to spinal surgeons for years.^[28]^

Diebo et al^[29]^ distinguished between patient-specific, non-modifiable risk factors (including age, BMI, and bone density), and surgically modifiable risk factors (surgical access, sagittal realignment, and junctional zone). We could not reproduce the surgically modifiable factors in full, but we took this into consideration during surgical planning and carried out revision surgery on three patients with osteoporosis. Contrary to some of the literature,^[4–6]^ we did not find that age, ASA score, smoking, localization, single-level or multilevel stabilization, or pathology were predictors of revision surgery. We used PEEK cages and autologous bone for fusion in all our patients. Therefore, we are not able to draw conclusions about the effect or not of PEEK cages on material failure.

In our study, BMI was a risk factor for revision surgery. Sing et al^[30]^ described obesity as an independent risk factor for early complications after revision spine surgery. A recently published study by Jain et al^[31]^ reported on the impact of obesity in patients undergoing elective spinal instrumentation; they found obesity to be an essential risk factor for the outcome of the primary surgery and for revision surgery as a complication (level of evidence 3).

The presence of radiolucent zones around the pedicle screws on follow-up CT scans is widely accepted as a definite indicator of postsurgical pseudarthrosis.^[32]^ Radiolucent zones do not necessarily involve instability. Tokuhashi et al^[33,34]^ reported that radiolucent zones around the screws disappeared over time in approximately two-thirds of patients treated with posterior instrumentation, and their presence did not necessarily indicate permanent pseudarthrosis or instability. In the present study, material failure occurred in 13 patients (8.5%), usually in the lumbar spine. Six of these patients showed an abnormality (radiolucent zone) in the CT monitoring scan, whereas the other seven presented complete material failure with no abnormalities at the 3 month follow-up.

Another potential reason for material failure is latent infection of the implant.^[26]^ Swabs were taken and the screws were sonicated during the surgical revisions to rule out a latent infection, and a superinfection of the material was found in only one patient.^[16,17,26]^

To the best of our knowledge, this study is one of the few that considers whether conspicuous image morphology is always an indication for early revision. Smith et al^[35]^ found a 6.8% incidence of rod fractures (i.e., material failure) after surgical treatment of patients with adult deformity, and a 15.8% incidence after pediculo-osteotomy. Several working groups describe different techniques to address and better understand this problem, such as using rods with a larger diameter, changing rods to cobalt-chrome implants, or applying double rods and multiple rods.^[21,28,36,37]^ Fortunately we had no problems with rods, perhaps because we always used the same system from the same company.

The timing of material failure must also be taken into account. The data in the present study show a peak in frequency in the first postoperative year, probably due to gradual material fatigue in the presence of instability. In Luca et al,^[38]^ material failure occurred on average after 11 months (range 8–15 months). However, other authors suggested scheduling a regular follow-up 6 months after the operation.^[4,9,10]^ We are considering introducing such an additional check-in future because we have observed that patients often report more pain between discharge and the 3-month follow-up than at the 12-month check.

In our experience, the three factors of increasing pain or high VAS score after surgery, non-fusion after three months, and a radiolucent zone or zones combined with material failure, are possible indications for early revision surgery.

Our recommendation meets the criteria for a level of evidence 3, based on the publication by Kaiser at al.^[39]^

### 4.1 Limitations

The limitations of this work are the retrospective study design, the non-homogeneous study population due to the heterogeneity of the underlying spine pathologies. Furthermore, follow-up may be necessary for more than 18 months. Moreover, the small number of patients limit the results, and we used PEEK cages for fusions below Th 4. Another important limitation of this paper is that we included different segments of the spine: cervical, thoracic, and lumbar.

## 5 Conclusion

With detailed knowledge of the timing of the material failure, the treating physician can provide optimal information to the patient. Radiological abnormalities alone (radiolucent zones) are not always associated with clinical worsening. Both the indications for early revision and the timing for surgery should be carefully assessed, and will depend on the level of experience at the particular clinic and the type of material failure.

## Author contributions

Conceived, designed, and performed the study: MB and HV. First drafting of the manuscript and illustrations: MB. Data acquisition: MB and JW. Analysis and interpretation of data: MB, JW, and HV. Critical review of the manuscript: JW, AS, GB, JS, and HV. The final manuscript was critically revised and approved by all authors.

**Data curation:** Abdallah Salemdawod, Gregor Bara.

**Formal analysis:** Johannes Wach.

**Software:** Abdallah Salemdawod.

**Supervision:** Hartmut Vatter.

**Validation:** Johannes Wach, Gregor Bara, Jasmin Scorzin.

**Visualization:** Jasmin Scorzin.

**Writing – original draft:** Mohammed Banat.

**Writing – review & editing:** Mohammed Banat, Hartmut Vatter.
